# Translating Building Information Modeling to Building Energy Modeling Using Model View Definition

**DOI:** 10.1155/2014/638276

**Published:** 2014-09-17

**Authors:** WoonSeong Jeong, Jong Bum Kim, Mark J. Clayton, Jeff S. Haberl, Wei Yan

**Affiliations:** Architecture Department, Texas A&M University, College Station, TX 77843, USA

## Abstract

This paper presents a new approach to translate between Building Information Modeling (BIM) and Building Energy Modeling (BEM) that uses Modelica, an object-oriented declarative, equation-based simulation environment. The approach (*BIM2BEM*) has been developed using a data modeling method to enable seamless model translations of building geometry, materials, and topology. Using data modeling, we created a Model View Definition (MVD) consisting of a process model and a class diagram. The process model demonstrates object-mapping between BIM and Modelica-based BEM (*ModelicaBEM*) and facilitates the definition of required information during model translations. The class diagram represents the information and object relationships to produce a class package intermediate between the BIM and BEM. The implementation of the intermediate class package enables system interface (*Revit2Modelica*) development for automatic BIM data translation into *ModelicaBEM*. In order to demonstrate and validate our approach, simulation result comparisons have been conducted via three test cases using (1) the BIM-based Modelica models generated from *Revit2Modelica* and (2) BEM models manually created using LBNL Modelica Buildings library. Our implementation shows that *BIM2BEM* (1) enables BIM models to be translated into *ModelicaBEM* models, (2) enables system interface development based on the MVD for thermal simulation, and (3) facilitates the reuse of original BIM data into building energy simulation without an import/export process.

## 1. Introduction

The exchange of data between building design representations and energy simulation representation has been a major challenge in the design process, resulting in the fact that building energy performance simulation is often omitted from the process [1]. The translation process is labor intensive, error-prone, and cumbersome [1–4]. Although tools have been developed to support the generation of an energy model from a design model, disconnections still exist between the various models [1, 5–7]. We speculate that many of the problems derive from building energy simulation tools that fail to take advantage of object-oriented programming (OOP) and do not easily allow for mapping from an object-oriented design model. To improve and enhance the model translation effectiveness, we investigated a new approach to link building information modeling (BIM), which is commonly used to support architectural design, to building energy modeling (BEM) that supports energy simulation. We used C# programming to directly access the object-oriented data representation within a BIM authoring system, and Modelica, an object-oriented physical modeling language to simulate the energy performance [8]. Our hypothesis is that use of object-oriented constructs within both BIM and BEM will enable more efficient and reliable translation and improve maintainability.

Our research has employed data modeling to develop* BIM2BEM* software to connect autodesk Revit to the Modelica Buildings Library [9]. In this paper, the* BIM2BEM* automatically generated BEM is called* ModelicaBEM. *It contains information derived from the BIM and can execute thermal simulation to obtain building performances such as indoor temperature and energy loads. We have developed* BIM2BEM* software to translate from the BIM to the* ModelicaBEM*. The objectives of our research are (1) to facilitate the reuse of original BIM data in building energy simulation without an import/export process; (2) to enable moderately complex multiple-zone BIM models to be translated into* ModelicaBEM*; and (3) to ease and facilitate further development through object encapsulation and provision of well-defined interfaces.

Our research process has been to use data modeling to create Model View Definitions (MVD) that consist of a process model and a class diagram and then conduct testing to assure that the translation works properly. We hoped to discover the effects and opportunities that arise. The following four phases have been conducted for the MVD development.

1. Develop a process model to document the mapping from BIM to BEM.
2. Develop class diagrams to represent the required information and object relationships.
3. Implement the translation classes to support BEM model creation using BIM data. We refer to this as the* BIM2BEM* software.
4. Conduct tests to demonstrate and validate the* BIM2BEM* approach.

The objectives of* BIM2BEM* are (1) to enable multiple-zone BIM models to be translated into* ModelicaBEM* models, (2) to enable system interface development based on the MVD for thermal simulation, and (3) to facilitate the reuse of original BIM data in building energy simulation without an import/export process.

The research scope is confined to translating the building envelope information of BIM, including geometry, material, and topology of a building model. In this paper, the Modelica-based BEM models translated from BIM models are called* ModelicaBEM* that contain BIM information and can execute thermal simulation to obtain building performances such as indoor temperature and energy loads. The terms “Building Information Modeling” or “Building Information Model(s)” are used interchangeably in different contexts and are abbreviated as BIM.

## 2. Background and Problems

Studies have presented the value of reusing data that has been produced by building designers when creating building energy models [1, 2, 6, 7, 10]. To increase the usability of data from designers in building energy simulation, various research prototypes [1, 2] and commercial products have been created, such as Green Building Studio.

However, reliably generating high quality BEM using current tools remains difficult. Although much of the process has been automated, intervention by the user to simplify models, choose among representations with subtle differences, and correct errors is still needed. For example, the users of Green Building Studio, which is a web-based energy analysis tool working with Revit (a BIM authoring tool developed by Autodesk), must finish the model check process to create a reliable gbXML file. Current energy simulation engines have their own unique input formats consisting of nonobject-oriented text files with highly specialized syntax and semantics [7]. The different data structures between BIM and an energy simulation engine often prevent efficient data translation or exchange. For instance, a translation process is required to perform data exchange through standard data schemas, which hinders the utilization of the parametric modeling capability of BIM in the design process. While a limited number of energy simulation tools support standard schema-based model translation, the absence of a standard interface in the tools also requires additional efforts and understanding of simulation processes for architects and designers to obtain building analysis results [7, 11, 12].

The efficient and effective data translation between BIM and building energy simulation can be achieved when two domains have the same modeling method such as an object-oriented method. A comprehensive data exchange model can then support direct mapping between them and facilitate an easy-to-use user interface implementation.

Based on the development of an interdisciplinary data exchange model and implementation of the model for direct mapping without an import/export process,* BIM2BEM* can facilitate the reuse of data from BIM in building energy simulation.

## 3. Research Objectives

This section describes challenges and tasks, tools and data, and methodology for* BIM2BEM* development. The* BIM2BEM* software is intended to handle the translation from a BIM to BEM represented in Modelica to facilitate executing a simulation with the Modelica Buildings toolkit.

### 3.1. Challenges

The main challenge of the project is to facilitate seamless model translation, requiring less manual data conversions between BIM and* ModelicaBEM*.

To achieve effective and efficient model translation, the following tasks need to be completed: (1) defining an object mapping process between BIM and* ModelicaBEM* to identify required information, (2) representing the identified datasets for the object mapping process, and (3) implementing the represented data subset and object relationships in a Modelica-based simulation tool.

#### 3.1.1. Define an Object Mapping Process between BIM and* ModelicaBEM*

Object semantics and relationships in architectural models are often represented differently than in the energy models. For example, in energy modeling building components are abstracted as 2D surfaces in order to enhance simulation performances, while the components are presented as 3D geometry in BIM.

To facilitate consistent object semantics and relationships between BIM and* ModelicaBEM*, an object mapping process needs to be conducted. The object mapping process demands to identify what information BIM and* ModelicaBEM* should be able to exchange. We utilized a data model method to classify mismatched object semantics and behaviors for the object mapping. The data modeling enables maintaining consistent object classifications of building components from BIM to* ModelicaBEM*.

#### 3.1.2. Represent Datasets for Object Mapping Processes

Different object semantics and relationships between BIM and* ModelicaBEM* demand their own data structure. For instance, data for building components such as walls, floors, and roofs are represented as 3D solids in BIM, whereas the same data are considered as surfaces in* ModelicaBEM*. In addition, a room object in BIM is represented as a zone in BEM, and the topology information for boundary condition is only represented in BEM, which can be retrieved by the combination of building objects information from BIM. To map the mismatched objects and behaviors, a data representation process is needed regarding what datasets in BIM and* ModelicaBEM* are used.

#### 3.1.3. Implement the Datasets and Object Relationships

The datasets and object relationships need to be created in a Modelica using parameters and functions. Instantiated objects can present building and related energy components of* ModelicaBEM*. For example, the area parameter can represent diverse geometry instead of just rectangular shape. Building topology in BIM can be mapped into* ModelicaBEM* topology, and calculated area information from BIM can be stored through a parameter.

### 3.2. Tools

For the* BIM2BEM* development, we used the BIM authoring tool Autodesk Revit and its application programming interface (API), and the LBNL Modelica Buildings Library [9].

#### 3.2.1. BIM Authoring Tool (Revit) and Its API

BIM supports three-Dimensional, semantically rich, and parametric modeling for design and construction during a building's lifecycle [13, 14]. BIM tools represent such a capability through their own data structure and implement the structure using specific database schema [13–15]. The BIM tools allow the databases to be represented as standard data models such as Industry Foundation Classes (IFC, a standard data schema for exchanging data among different applications) through user commands or API [16–18]. Software developers can access specific building component data of Revit and create a comprehensive database through API using the C# language [19]. In our project, instead of using standard data models such as IFC or gbXML, we utilized the Revit API capability to access the BIM data directly to (1) preserve object relationships established by parametric modeling, (2) define a model view of Revit to support bidirectional data exchange with the object-oriented simulation solver—LBNL Modelica Buildings Library.

#### 3.2.2. Modelica and Dymola

To support modeling and simulation from a physical point of view, object-oriented physical modeling (OOPM) has been developed to offer a structured and equation-based modeling approach [20, 21]. Modelica is an OOPM language and enables users to model the complex design of mechanical, electrical, and control systems using differential algebraic equations of relevant physics laws [21]. Modelica can represent topology of energy models using components and object connection diagrams [21]. Such capabilities can facilitate an object mapping from the BIM structure to* ModelicaBEM* naturally. Modelica libraries such as LBNL Modelica Buildings Library [9] facilitate the use of Modelica in thermal simulation, offering model components and solvers. We used Dymola [22] as an integrated simulation environment for Modelica models with LBNL Modelica Buildings Library as the thermal simulation engine.

#### 3.2.3. LBNL Modelica Buildings Library

The LBNL Modelica Buildings library has been developed for building energy simulations to support the simulation of heating and cooling system, controls, heat transfer through building envelopes, and airflow [23]. One of the major resources for building thermal analysis in the library is the HeatTransfer and Room packages, which have been validated through benchmarked simulation models [24, 25]. The validation accounts for the capability of whole building simulations [24].

In order to create* ModelicaBEM* that can use the LBNL Modelica Buildings Library for building thermal simulation, Modelica code must be created based on BIM data. Insufficient data exchange capability between BIM and the library results in the designers' subjective interpretations of building data and human errors in creating* ModelicaBEM*. In addition, the absence of a de facto standard interface for the data exchange causes a difficulty in translating BIM into* ModelicaBEM* incorporating with the library.* BIM2BEM* facilitates the data exchange through the model view of the library and the intermediate classes.

### 3.3. Methodology and Tasks

Our methodology in the* BIM2BEM* development includes (1) developing an MVD through data modeling, (2) implementing the designed classes in the MVD using the Modelica and the C# language, and (3) conducting test cases for validation by simulation of result comparisons for multizone models.

#### 3.3.1. MVD Development

We utilized a data modeling method to develop an MVD for data exchange between BIM and* ModelicaBEM*. The MVD consists of (1) modeling the process to map objects and overcome mismatched objects' semantics and behaviors and (2) designing classes to represent the required information and object relationships. 

*(1) Process Modeling*. The process of interest in our research is the mapping from BIM to BEM. We used process modeling to identify required information and object relationships. Although some data can be easily translated, the challenges arise from recognizing mismatched object semantics and behaviors between the BIM and the BEM. From the investigation of architectural modeling and building energy modeling, we can distinguish the mismatches into (1) semantic mismatches of building components and (2) behavior mismatches between BIM and BEM. Resolving these mismatches was a major task in this research.

Semantic mismatches hamper data exchange of objects and parameters because the starting representation and the ending representation make use of fundamentally different abstractions. For example, BIM represents a building envelop by composing building components such as walls, floors, and roofs, while BEM represents the envelope as exterior and interior surfaces. In order to map the building components into exterior surfaces, the required information can include the area of the surfaces and the summation of them through a function. To implement the function, the object relationships between related building components need to be defined to inform what kind of and how many surfaces constitute the whole exterior surface.

Behavior mismatches occur when the objects are similar or identical in BIM and BEM, but the behavior of the object is different. The required information must be derived by applying a rule that accepts the BIM information as input and produces the BEM information as output. For example, when a user separates two rooms by using an interior wall in BIM, BEM defines the boundary conditions to facilitate heat transfer between the separated rooms. We can establish a rule: if one surface of the wall object in BIM is defined as a* surface boundary*, the other surface can be a* construction boundary* automatically to map the boundary condition into BEM. BEM defines the two boundary conditions to calculate heat transfer on interior walls between thermal zones. We can apply the rule in generating* ModelicaBEM*'s building topology. The boundary condition information can be obtained by implementing the rule using a room object and building components enclosing the room such as walls, floors, and roofs.

The process modeling method involves decomposing the process into a series of activities, connecting them into a logical sequence, and collecting the data requirements [26, 27]. We used process modeling to identify the required information and object relationships and support more efficient workflows [28]. The process model can be then used in defining the scope of data modeling.

There are several graphic and nongraphic methods for process modeling, such as the Flowchart, unified modeling language(s) (UML), and IDEF0 [29]. IDEF0 (integrated definition of functional modeling) is most commonly used in product data modeling [30]. We used IDEF0 to describe how activities for the mapping are connected, ordered, and structured. The unique feature of IDEF0 models is its ICOM codes (Input, Control, Output, and Mechanism presented by arrows): Input and Output arrows represent the data and object flows into and out of a function; Control arrows indicate the required conditions for a function; and Mechanism arrows denote the means to performing a function [31]. IDEF0 models are especially useful in understanding a data flow [32]. We created an IDEF0 diagram for the process model and then defined additional information that is needed to map data between BIM and* ModelicaBEM*. The information will be represented through a class diagram including attributes and class relationships.  Section 4 explains how requirements for object mapping can be represented using IDEF0 specifications.

*(2) Class Design.* We developed a class diagram to represent specific data types and object relationships as objects and relationships. Based on the investigation of the mapping process for the required information and object relationships, we created two model views to define datasets: Revit Model View and Modelica Model View. Based on the two model views, we created an intermediate class package consisting of wrapper classes and interface classes as an Exchange Model View.

The class diagram enables* ModelicaBEM* not only to follow the data structure and semantics of BIM but also to represent related information for thermal simulation. The following section describes how to create the class diagram using UML.

#### 3.3.2. Implementation

We used the C# language to implement the functions in the interface classes, which facilitate data transformations such as building topology translation.

We used wrapper classes in Modelica to bridge between the Revit BIM classes and the energy model classes in the LBNL Modelica Buildings library.

The wrapper classes enable* ModelicaBEM* to populate instantiated objects. Consequently, a* ModelicaBEM* is able to represent mismatched semantics and behaviors by composing related instances and parameters that store the values from BIM.* ModelicaBEM* rely on a system interface that can preprocess BIM to prepare the required information and assemble the instantiated objects before the* ModelicaBEM* reaches the LBNL Modelica Buildings Library. The interface classes enable* Revit2Modelica* to preprocess BIM.

#### 3.3.3. Conducting Test Cases and Simulation Result Comparisons

Three test cases have been studied to demonstrate and validate the* BIM2BEM *approach. For demonstration, a prototype shows how multi-zone BIM models can be automatically translated into* ModelicaBEM*. In case of validation, the simulation result comparisons are conducted between two BEM models in each test case: one is automatically generated* ModelicaBEM* and the other is the manually created model following LBNL's BEM structure.

## 4. *BIM2BEM* Development

BIM and LBNL's BEM (manually created using Modelica) follow object-oriented modeling concepts; however, they have different object semantics and behaviors, which are challenging for model translation. In this project, we developed an MVD to define data exchange requirements for Revit and the LBNL Modelica Buildings Library.

MVD usually defines the subset of IFC models for supporting data interoperability [33]. We adopted the concept of MVD to reduce the interoperability problem and support more seamless translation between BIM (Revit) and* ModelicaBEM*. The MVD development follows a modeling-diagramming-implementing approach: (1) developing a process model to identify building objects and their relationships, (2) creating a class diagram based on application model views and the exchange model view, and (3) implementing the intermediate class package.

### 4.1. Develop a Process Model for Translations

**Figure 1:**
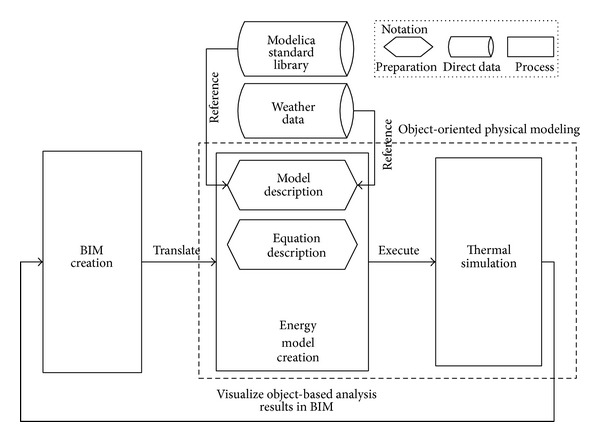
The overall translation process between BIM and* ModelicaBEM*.

To identify mismatched objects semantics and behavior, we studied the translation process between BIM and* ModelicaBEM*.  Figure 1 shows the overall process of how BEM is incorporated with BIM: data from BIM are translated into* ModelicaBEM*, and* ModelicaBEM* produces object-based results after completing the simulation, which are able to be displayed in BIM finally [34].

The data translation mainly occurs between BIM Creation and Energy Model Creation shown in Figure 1. The model description preparation in the Energy Model Creation activity is to populate required information from the Modelica standard library and the weather data. The major classes and parameters in LBNL Modelica Buildings Library are shown in Table 1. The boundary condition definition in the library has five types: exterior opaque surfaces (datConExt), exterior opaque surfaces with windows (datConExtWin), interior walls between thermal zones (datConBou or surBou), and interior partitions in a thermal zone (datConPar). We applied the translation rules (in Section 4.3) for translating the building topology into the boundary conditions.

The building can be represented with instances from the classes in Modelica using LBNL's library. As shown in Algorithm 1, a thermal zone consisting of six surfaces without any openings can be declared as a thermal zone instance (line 1) and surfaces information is given parameters of the zone instance. Six surfaces of the thermal zone are categorized as opaque surfaces (line 5), and their layer information (line 7), area (line 8), a tilt angle (line 9), and an azimuth angle (line 10) are provided.

LBNL Modelica Buildings Library is developed based on engineering-semantic point of view. As shown in Algorithm 1, the thermal zone instance does not require any wall instances to simulate a room model even though the building consists of several walls. Such mismatched object semantics (compared to BIM) require us to define an object semantics rule set to represent information in BIM for the BIM-to-*ModelicaBEM* translation. Based on the investigation and the guideline for object mapping [35], we set the rule set as follows.

- *Addition*: adding missing data in BIM that are required for* ModelicaBEM*, such as solar and infrared absorptivities, solar transmittance, and infrared transmissivity of glass.
- *Translation*: data translation between BIM and* ModelicaBEM* to represent mismatched semantics, such as rooms to thermal zones.
- *Calculation*: calculating new values using existing values in BIM, such as window-frame ratio and construction boundary types.

**Figure 2:**
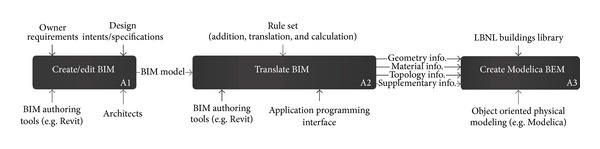
A process model of BIM-to-*ModelicaBEM* translation using IDEF0.

We can identify required processes applying the rule set to* ModelicaBEM* creation and represent the BIM-to-*ModelicaBEM* translation using IDEF0 as shown in Figure 2.

**Figure 3:**
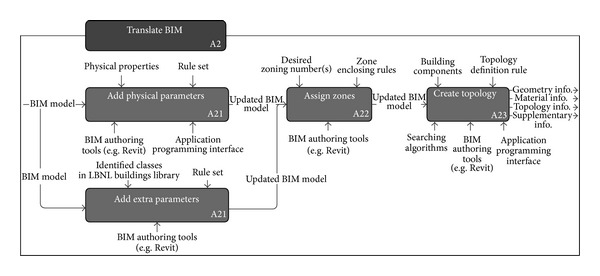
Detailed process model of translate-BIM activity to represent required data flow.

However, the Translate BIM activity only represents object semantics. As described in Section 3.3; the LBNL Modelica Buildings Library also represents object behaviors including building topology differently. To represent such mismatched behaviors, we identify detailed processes of the Translate BIM activity as shown in Figure 3.

Figure 3 shows the data flow of the Translate BIM activity including topology creation. The information in each step is considered as the requirements for the data model and they will be made into classes. Based on the processes and a mapping guideline [35], we defined the requirements from each step as shown in Table 2.

The data requirements in Tables 1 and 2 can be represented as classes and properties in a class diagram. The class diagram will also represent object behaviors via defined functions and relationships among classes.

### 4.2. Create Class Definitions

The class diagram contains classes, including properties and functions, and class relationships in application model views and the exchange model view.

The model views represent what the specific data and datasets are for the data requirements from the process models in Revit and LBNL Modelica Buildings Library (Revit Model View and Modelica Model View). The Exchange Model View consists of wrapper classes and interface classes and allows* ModelicaBEM* to hold building geometries, material properties, and topology for thermal simulation.

#### 4.2.1. Application Model Views

**Figure 4:**
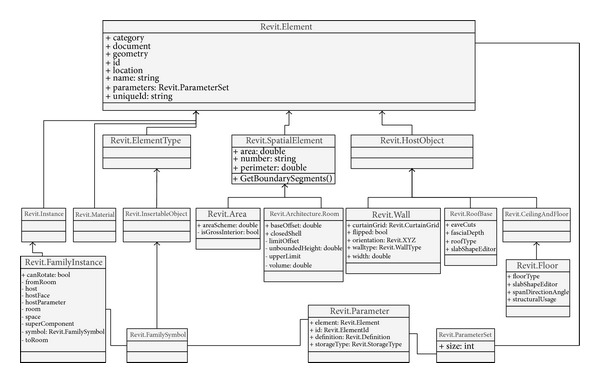
Class diagram of the Revit model view.

*(1) Revit Model View*. Revit models contain architectural data created by the users. Very large data sets can be represented in BIM; however, only part of the data is applicable to thermal simulation. The data for thermal simulation are represented as native Revit instances of classes and relationships shown in Figure 4. These classes can represent the data requirements categorized in Table 2. The description of the classes is adapted from Autodesk references [19]. Based on the description, we created a class diagram (Figure 4) using a UML Class Diagram to show the relationships among the classes [36].

- *Revit.Element*: the* Element* class represents geometry information of building components such as area, height, length, and volume through relationships with* Revit.Parameter* class. Such inherited classes from the* Element* class, for example,* Wall, Floor*, and* Roof (Base)* can have the geometry information.
- *Revit.Wall*: the* Wall* class, derived from the* Element* class, has additional geometry information derived from the* Element* class such as orientation and width information. Also, the type information of a wall can be defined through* WallType* class.
- *Revit.RoofBase*: the* RoofBase* class provides additional information such as the roof types. The type property can categorize roof instances with specified purposes such as flat roof or slope roof.
- *Revit.Floor*: the* Floor* class is inherited from the* CeilingAndFloor* class that provides support for all ceiling and floor objects including geometry information. This* Floor* class has an additional type property representing floor types.
- *Revit.FamilyInstance*: the* FamilyInstance* represents a single object of a family type such as doors and windows. If a door is created in a wall between two rooms, the connectivity information regarding which room is connected to another can be defined via the properties,* fromRoom* and* toRoom*. The* FamilyInstance* has a relationship with* FamilySymbol*, which enables additional parameters to represent thermal properties such as the frame ratio information. For example, a window family allows creating a thickness parameter to calculate the frame ratio. The parameters are represented through the* FamilySymbol* class, and the* FamilyInstance* class represents a window object.
- *Revit.Architecture.Room*: the* Room* class represents the basic information such as area, height, and perimeter inherited from the superclass (*SpatialElement*). The volume information of a room is only defined in the* Room* class. Such a relationship provides access to retrieve required information after a room instance is created.
- *Revit.Material*: the* Material* class represents material information regarding the color of the material, the name of general material types, the shininess and smoothness of the material, and so on. The category property in this class enables building components to access their material information, that is, once a wall instance is created, the instance has parameters including a material instance.

Using the objects described in this class diagram, the steps of “Create/Edit BIM,” “Add extra parameters,” and “Assign zones” in the process model can be defined. “Add physical parameters” can be composed through functions in the interface classes.

*(2) Modelica Model View*. We created a Modelica model view to represent the information identified in Table 1. The model view shows the classes and relationships that are defined to create a model using the LBNL Modelica Buildings library. The classes are MixedAir, GlazingSystem, DoorDiscretizedOpen, FixedBoundary, WeatherData, and additional Modelica standard classes.

**Figure 5:**
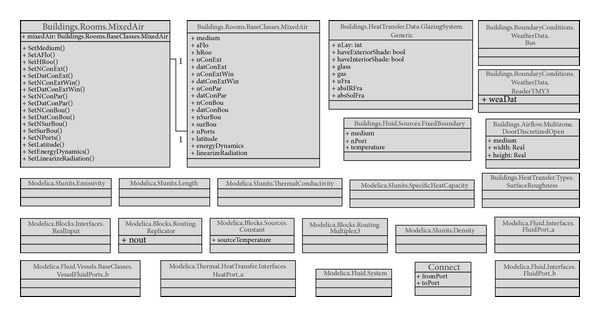
Class diagram of the Modelica model view.

Figure 5 shows the data subsets in LBNL Modelica Buildings Library as classes and relationships. For example, in the model description code block (Algorithm 1), the mixedAir object is instantiated from the MixedAir class under the Rooms package (Rooms.MixedAir), and the properties in the object are declared in another MixedAir class under the BaseClasses (BaseClasses.MixedAir). LBNL Modelica Buildings library defines the relationship between the two classes: the BaseClasses.MixedAir object is encapsulated as a parameter object in the Rooms.MixedAir object.

We used the UML specifications to represent the classes in Figure 5 based on our investigation of LBNL Modelica Buildings Library.

#### 4.2.2. Exchange MVD

We created an Exchange MVD to define interface and wrapper classes. The Exchange Model View integrates two different semantic models, which represent not only architecture but also engineering points of view. For example, the wall class has a material instance parameter and a different material type in the Modelica model, and the property of the wall instance such as area can be passed to the Modelica model. The room class has wall instances to pass material and area information to parameters in MixedAir class.

*(1) Wrapper Classes*. Wrapper classes allow* ModelicaBEM* models to follow object semantics of Revit and to utilize LBNL Modelica Buildings library. Wrapper classes adopt the following rules, which represent a common object relationship among building objects.

- A building object in Revit consists of room objects, walls, floors, and roofs enclosing the room.
- A room object in Revit is transformed into a single thermal zone object (MixedAir object in LBNL's BEM). A multi-zone model can be created through connecting multiple room objects.
- Topology for energy modeling can be translated from the connectivity of the Revit building components.

Based on the rules, wrapper classes include* Wall*,* Floor*,* Roof*,* Window*,* Door*, and* Room* classes. The instances from the classes enable the building component information to be transformed from BIM to* ModelicaBEM*.

**Figure 6:**
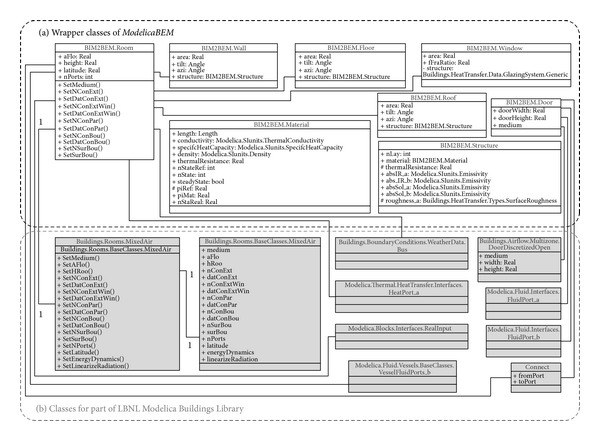
Class diagram of the wrapper classes.

Figure 6(a) shows the wrapper classes containing a series of properties that can transfer Revit parameters into* ModelicaBEM*. The relationships represent the rules. For example, the* BIM2BEM.Room* class has relationships with* BIM2BEM.Wall*,* BIM2BEM.Floor*, and* BIM2BEM.Roof* classes to represent the composition of a building object.

To differentiate class names between the application model views and wrapper classes, we specified the class name by starting with domain name; for example, the material class names in Revit and the wrapper classes are* Revit.Material* and* BIM2BEM.Material*, respectively. The wrapper classes are described below.

- *BIM2BEM.Room*: the* Room* class represents a single-zone model and wraps the* MixedAir* class of LBNL Modelica Buildings library in it. The MixedAir class models a room filled with mixed air. The MixedAir model and the library have been validated [9, 24, 25, 37]. The class properties and functions enable* ModelicaBEM* to populate required information such as building materials and components and thermal boundary conditions. For example, area, tilt, and azimuth as parameters are created in a room object.
- *BIM2BEM.Wall*,* BIM2BEM.Floor*, and* BIM2BEM.Roof*: these classes store the basic building component information from BIM including area, tilt, azimuth, and material layers for energy modeling. The material layer information is represented as a parameter of a* BIM2BEM*.*Structure* instance. The defined relationships between the Room class and the building components classes enable each instantiated building object to be encapsulated in a room object to pass the geometry information.
- *BIM2BEM.Window*: the* Window* class represents window geometry data as well as the data of glass panels and window frames in the Modelica model view. The* Window* class can represent material information by defining GlazingSystem.Generic instance as a parameter.
- *BIM2BEM.Door*: the* Door* is a wrapper class of the* DoorDiscretizedOpen* class and consists of geometry properties such as width and height for calculating multizone airflow. Currently, we implemented a closed door to calculate heat transfer and infiltration through the door. A port property represents room-door-room connection as a parameter.
- *BIM2BEM.Structure* and* BIM2BEM.Material*: the* Structure* and* Material* classes are designed to represent material properties and geometry information of opaque constructions and how materials are assembled. The* Structure* class contains construction information with the number of layers where each layer is represented by* Material* instances. The Material class represents thermal information such as thermal conductivity, heat capacity, and density. The additional thermal properties for inner and outer surfaces such as solar and infrared absorptivities exist in the* Structure* class.

**Figure 7:**
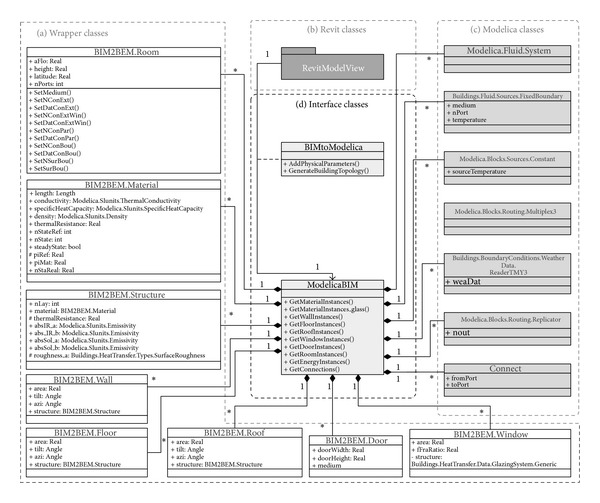
Class diagram of the interface and wrapper classes.

*(2) Interface Classes*. The interface classes are as shown in Figure 7.

The methods in the interface classes enable* ModelicaBEM* to contain object instances following Modelica language specifications. The following classes present the functions and object relationships in* ModelicaBEM*.

- * ModelicaBIM*: the* ModelicaBIM* class has functions to populate a* ModelicaBEM* model, which consists of three parts: building model description, energy components, and connections. The relationships in Figure 7 show the composition (e.g., the building model description is represented with composition relationships among the classes in the wrapper classes).

The functions enable the* ModelicaBEM* objects to instantiate the classes. For example, the* GetMaterialInstances()* function instantiates the* BIM2BEM.Material* class to present related materials in the building model description. As shown in Figure 7(d), eight functions map building envelope data, boundary conditions, and room geometry information from Revit to wrapper classes. In addition, two functions are defined to describe energy components and connections. For example, the energy object from the* WeatherData* class provides selected weather information to the* ModelicaBEM*; the information of selected geographical location is retrieved from Revit by the user's setting of the building location.

- * BIMtoModelica*: the* BIMtoModelica* class has two functions to support the* ModelicaBIM* class having relationships with Revit classes as shown in Figure 7(b). The two functions are implemented using Revit API. The* AddPhysicalParameters()* function allows a Revit model to include missing physical properties defined in Table 2. The specific values of boundary conditions in a Room instance can be computed through the* GenerateBuildingTopology()* function, which provides the boundary condition type information and generates building topology from an extended BIM.

A detailed example of creating* ModelicaBEM* through the interface classes will be explained in the following section.

### 4.3. Implement Wrapper and Interface Classes

*ModelicaBEM* can be created with the implementation of the classes of the wrapper and interface.

The implementation of the wrapper classes is accomplished using Modelica language specifications.  Algorithm 2 shows the implementation consisting of four declaration sections: Property, Wrapper component, Energy component, and Connect declaration. The Property declaration demonstrates how defined properties in* BIM2BEM.Room* class can be implemented using Modelica. The Wrapper and Energy component declarations represent the object relationships between the Room class and the connected classes. The Connect declaration implements connectors that represent a physical flow between the wrapper components and the energy components. Based on the implementation, the class package can act as a template when creating new instances.

The interface classes' implementation facilitates a system interface to automatically create the required instances of* ModelicaBEM*. Based on the class specification, we developed the* Revit2Modelica* prototype to transfer data from Revit into* ModelicaBEM*. The prototype enables a Revit model to contain additional materials and define building topology for* ModelicaBEM*. For example, the prototype implements the* GenerateBuildingTopology()* function to map construction boundary condition types into* ModelicaBEM*. The boundary condition types are calculated based on our predefined rules as follows.

- Exterior walls, roofs, and floors, which enclose a room and have nonsharing building components with any other rooms, should be mapped into opaque surfaces.
- Exterior walls enclosing a thermal zone and containing a window(s) should be mapped into opaque surfaces with windows.
- Shared interior walls between rooms should be defined as opaque surfaces on the location of the interior walls as construction boundaries and surround boundaries between thermal zones.
- Interior walls inside a single room should be mapped into interior partitions in a thermal zone.

In addition, object instances can be generated through the* Revit2Modelica* prototype as shown in Algorithm 3.

The Exchange MVD enables the object mapping between the two applications based on the object-oriented modeling concept. In addition, the demonstration shows that the MVD follows the Object-Oriented Programming (OOP) approach in the development, which facilitates natural object mapping between Revit and LBNL Modelica Buildings library. Algorithm 3 shows the encapsulation characteristic of the OOP approach; once the required instances are populated from the prototype, the values of them or instances themselves are encapsulated in other instances. As an example of material objects, the material object of a wall is encapsulated as a parameter object in a wall instance to represent the material information.

## 5. Experiments

To validate the* BIM2BEM* approach, we conducted (1) experiments by applying the class package and* Revit2Modelica* to multizone BIM and (2) simulation result comparisons between two Modelica models: one generated automatically using* Revit2Modelica* and the other manually following the LBNL Modelica Buildings Library samples' approach. For the experiments, three test case models are used. We hypothesized that if our exchange MVD represents all requirements, and if the implementation of the MVD is accurate, the two Modelica models of each test case can produce close or identical simulation results.

### 5.1. Test Cases

For the test cases, we created corresponding BIM models using Autodesk Revit Architecture for: a two-room model (Test Case 1), a two-room model having two windows and an interior door (Test Case 2), and a two-story model having two windows (Test Case 3). Test Case 1 has two thermal zones, six exterior surfaces, and one interior wall. Based on this test case, other models are created to present more building components such as windows, doors, and a new story. The following sections discuss the test cases.

#### 5.1.1. Test Case 1: Creating a Basic Building Model with Two Thermal Zones

**Figure 8:**
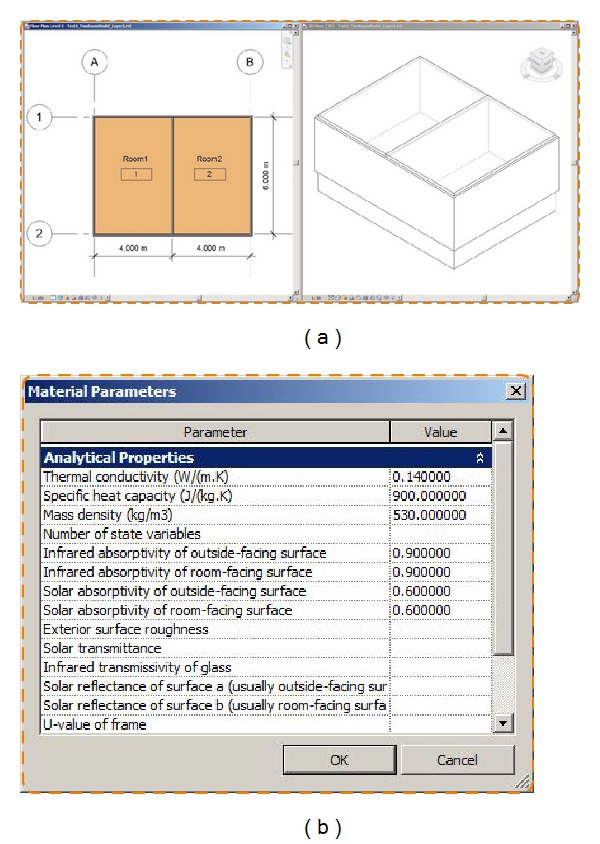
A two-thermal-zone Revit model. (a) Floor-plan and isometric views. (b) The custom parameter window for adding additional physical properties.

Test Case 1 presents a two-thermal-zone model without windows and doors. The building dimensions are 8.0 m∗6.0 m∗2.7 m, respectively, (Figure 8). The basic material information is defined in Table 3.

To add required physical properties in Table 3, we used the* AddPhysicalParameters()* function as shown in Figure 8(b).

The* Revit2Modelica* prototype translates the two-room Revit model into a* ModelicaBEM*. Algorithm 3 shows the generated instances for* ModelicaBEM* from the* Revit2Modelica* prototype. The* GetMaterialInstances()* function in ModelicaBIM class can collect each material instance information used in a BIM model following the Material class of the implemented class package. Wall material objects are instantiated and values for physical properties, which are prepared through the custom parameter window, are assigned to the parameters. Then, the wall objects are instantiated based on the geometry information and the material instances.

The* GenerateBuildingTopology()* function in the* BIMtoModelica* class can retrieve the values of the boundary conditions. As shown in Algorithm 3, the left room has five opaque surfaces (nConExt and datConExt) for three exterior walls, a roof, and a floor, and one opaque surface that is for the interior wall between the thermal zones (nConBou and datConBou); the right room has one opaque surface on the same interior wall between the thermal zones (nSurBou and surBou). The interior wall instance is used in each room (Room instances in Algorithm 3).

#### 5.1.2. Test Case 2: Adding Windows and an Interior Door

**Figure 9:**
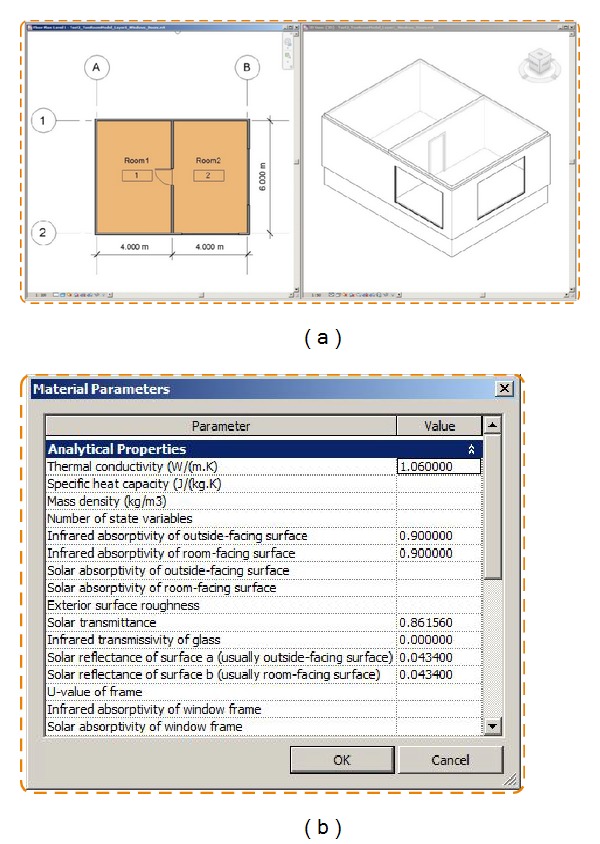
A two-thermal-zone Revit model with two windows and a door. (a) Floor-plan and isometric views. (b) The custom parameter window for adding additional physical properties for the glazing system.

We expanded the two-thermal-zone model by installing two windows on the south and east exterior walls, respectively, and a door in the interior wall as shown in Figure 9(a). The size of each window is 6 m^2^, and the additional physical material information for the glazing system defined in Table 2 is prepared through the custom parameter window (Figure 9(b)).

**Figure 10:**
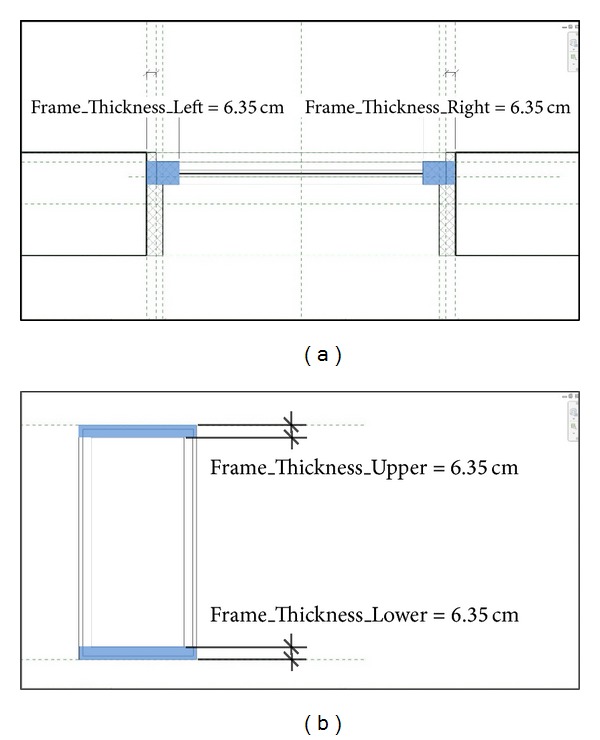
The window family in Revit, (a) a plan view and (b) an elevation view.

To prepare the additional thermal information for the glazing system such as the ratio of window frame, we created new parameters in the existing Window family in Revit (Figure 10). The calculated value of the window frame ratio is used as a parameter of a window instance in the* ModelicaBEM* (fFraRatio of the Window instance in Algorithm 4).

By installing two windows in the right room, the room has three opaque surfaces (nConExt and datConExt in Room2 of Algorithm 4) and two opaque surfaces with windows (nConExtWin and datConExtWin in Room2 of Algorithm 4).

The line of the Modelica code for a door object is generated through* Revit2Modelica* (the Door instance in Algorithm 4) following the Door class definition in the wrapper classes (Figure 6). By wrapping a Door class, two Modelica* connects* need to be created to calculate bi-directional airflow between two the rooms. Therefore, four Modelica connects are created to link the door and two rooms (Connect instances in Algorithm 4).

#### 5.1.3. Test Case 3: Adding a New Story

**Figure 11:**
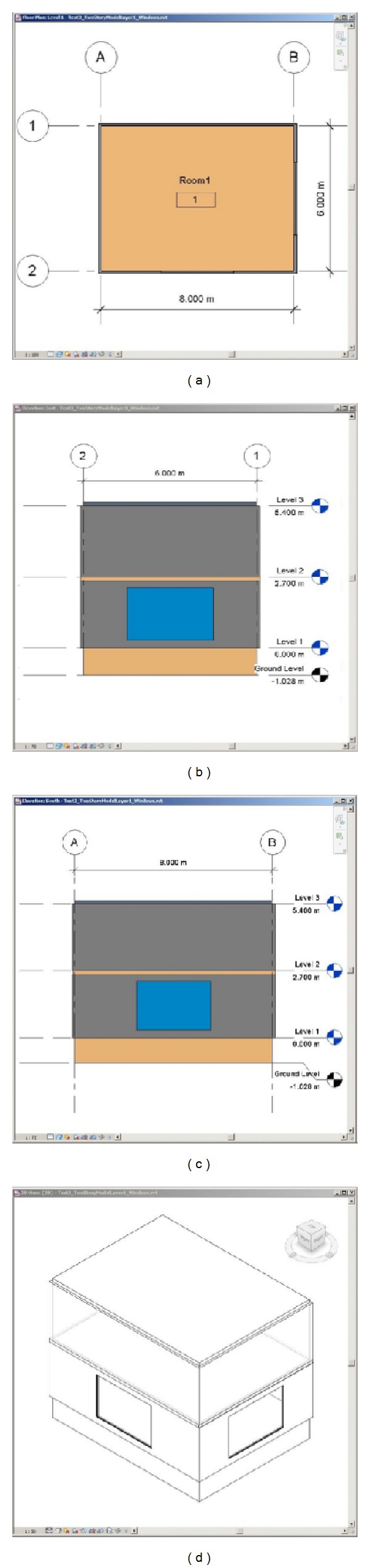
A two-story Revit model. (a) Floor-plan view of first level, (b) East-elevation view, (c) South-elevation view, and (d) isometric view.

To demonstrate a zoning case for vertical stacking of rooms, we created a two-story Revit model as shown in Figure 11. The building has one room on each floor and a 6 m^2^ window on each of the south wall and the west wall at the first floor.

Based on the BIM of the building,* Revit2Modelica* creates* ModelicaBEM* that enables heat transfer to be simulated between two stories. The mechanism of the heat transfer is similar to Test Case 1. The major difference is that the floor object, instead of the interior wall, connects two thermal zones.

The roof instance in the lower level and the floor instance in the upper level are modeled in the* ModelicaBEM* as opaque surfaces (the Floor instance and the Roof instance in Algorithm 5). Then, a Modelica* connect* is created to link the opaque surfaces for conduction heat transfer calculation.

In terms of boundary conditions, the right room of Test Case 1 and the upper room in Test Case 3 have the same number of opaque surfaces (nConExt and datConExt) and another opaque surface between two thermal zones in each case (nSurBou and surBou). In Test Case 3, the five opaque surfaces in the upper room consist of four walls and a roof, and the one opaque surface between the lower room and the upper room is the floor object (Room instance in Algorithm 5).

As shown in Algorithms 3, 4 and 5, the generated three* ModelicaBEM* of the three test cases demonstrate the use of the* Revit2Modelica* prototype. To validate the method and the prototype, we conducted simulation result comparisons explained blow.

### 5.2. Simulation Result Comparisons

We utilized Dymola as a Modelica development environment and LBNL Modelica Buildings library version 1.3 to perform thermal simulation with the* ModelicaBEM*. As Modelica Buildings requires designation of time intervals and tolerances, the simulation settings include time interval of 3600 seconds for a one-year period and a tolerance of 10^−6^.

We applied the consistent model conditions for all the building models as follows.

- The floor is above the ground level.
- Each room is a single thermal zone.
- The building location is Chicago, Illinois, USA.
- The building has no shading devices and no internal heat gains from equipment and occupants.
- The building has no HVAC systems.
- The windows and the door are closed.

The simulation results of each test case model agree with the results of each of the corresponding model created manually using the LBNL Modelica Buildings library sample structure by us, in terms of annual indoor air temperature and heat flow.

The indoor air temperatures of each Modelica model in the test cases are almost identical with those of LBNL's models. For example in Test Case 2, as shown in Table 4, the highest temperatures are obtained at 12PM on August 21th in East Rooms in both models, and in Test Case 3, the lowest temperatures are obtained at 8AM on January 8thin the Upper Rooms in both models.

**Figure 12:**
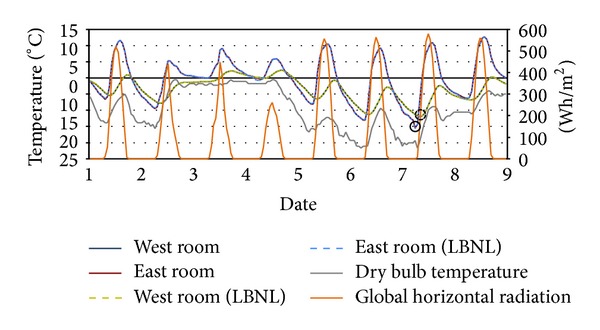
Temperature comparison of Test Case 2 for February 1st to 9th. Solid (6AM, February 7th) and broken (8AM, February 7th) circles point out the lowest temperature in the East and West Rooms, respectively, for the two models. The temperature curves overlap between the two models.

**Figure 13:**
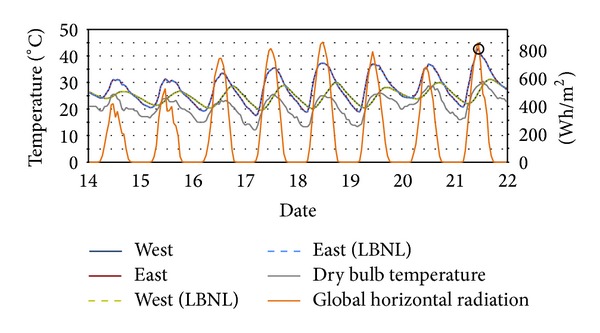
Temperature comparison of Test Case 2 for August 14th to 22nd. Solid circle (12PM, August 21st) points out the highest temperatures of the East Room. The temperature curves overlap between the two models.

**Figure 14:**
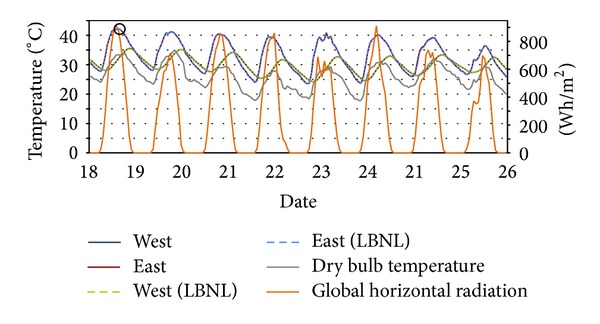
Temperature comparison of Test Case 2 for July 18th to 26th. Solid circle (6PM, July 18th) presents the highest temperatures of the West Room. The temperature curves overlap between the two models.

Figures 12, 13, and 14 show the results in Test Case 2: the indoor air temperatures of* ModelicaBEM* and LBNL's model, global horizontal radiation, and a dry bulb outdoor temperatures during different time periods and for different rooms from February 1st to 9th, from July 18th to 26th, and from August 14th to 22nd respectively.

**Figure 15:**
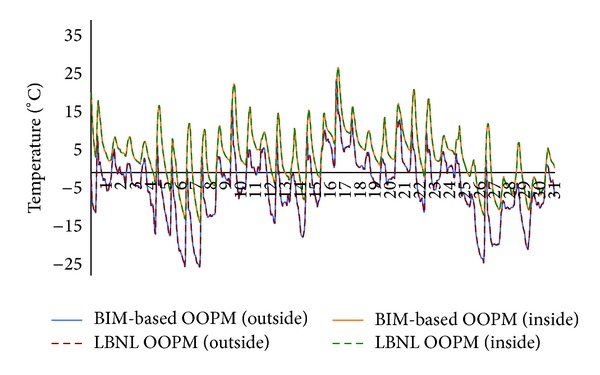
Temperature comparison between the two models for the east wall in Test Case 2 during January.

**Figure 16:**
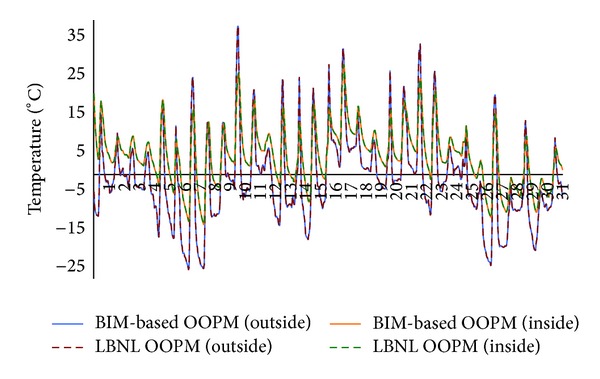
Temperature comparison between the two models for the south wall in Test Case 2 during January.

We also conducted a validation case study for component level analysis. We examined the temperatures from outside surfaces and temperatures of the inside surfaces of the east and south walls each having a window in Test Case 2. As shown in Figures 15 and 16, the temperature graphs of* ModelicaBEM* almost overlap those of LBNL's model.

Overall, the BIM-based* ModelicaBEM* models created by our prototype produce very similar simulation results as LBNL's models. This is expected because the same thermal simulation algorithm provided by LBNL Modelica Buildings library is applied to all the energy models.

The modeling method (automatic versus manual) and model structures (one is BIM-based and the other is not) are the major differences between* ModelicaBEM* and the LBNL's models. The simulation results will have inconsistency during the comparison studies if the model translations were not done correctly by* Revit2Modelica*.* Revit2Modelica* generates the energy models that are reasonably more comprehensive to reflect the actual building configuration. In the example of Test Case 3, the LBNL's Modelica model represents the floor object as a roof object for the lower thermal zone and a floor object for the upper thermal zone. Based on the understanding of energy semantics, for example, boundary condition, the shared floor object is modeled into thermal zones manually. However, our approach automatically splits the floor object as two components to represent the actual building configuration: one is for a floor object in the upper room and the other is for a roof object in the lower room.

In terms of the model structure,* Revit2Modelica* generated energy models present to the user architectural semantics such as rooms, instead of energy engineering-based semantics such as MixedAir. In addition, the two structures can have a difference regarding the order of building enclosure elements used as arguments in thermal calculation functions, causing the slightly different simulation results. The result differences due to this argument order difference can be reduced through decreasing the model tolerance value in Dymola.

## 6. Conclusions and Future Work

This paper presents a translation method for integrating BIM and OOPM (Modelica) for building energy simulation. Our* BIM2BEM* development enables interdisciplinary data exchange between architectural design and building energy simulation.* BIM2BEM* can leverage the consistent use of the architect's data (such as the building geometry, materials, and even parametric objects) in building energy simulation without recreating them in energy models manually. Reuse of the data from BIM can significantly reduce the effort required for the definition of input data in BEM. The process presented in this paper has the potential to eliminate error-prone manual processes.

Our data modeling approach facilitates the development of a system interface for automatic translation from BIM to BEM with high efficiency and accuracy. While the file-based translation through standard schema such as IFC and gbXML can often facilitate the translation between different BIM tools and different simulation applications, implementing the complex schemas of IFC demands enormous amount of time and efforts [38]. The developed prototype based on the Exchange MVD enables more seamless design-simulation integration while the BIM tools (such as Revit) can preserve the parametric modeling capability in the process. The current version of the MVD is applicable for Revit; however, the MVD and the system interface can be developed to support other BIM tools such as ArchiCAD, AECOsim Building Designer V8i, and Allplan. Nevertheless, the use of IFC can better bridge between multiple BIM authoring tools and diverse simulation tools.

The process for assigning additional physical parameters is semiautomatic: users are expected to assign the values of the parameters manually, but the parameters are created in BIM automatically. In the future development, this process can be fully automated by linking the material parameters to existing material database in order to retrieve the parameter values. In the present system, after a complete BIM model is created with all required information, the translation process is automatic.

As an application, the developed system interface supports object-based thermal performance results to be displayed using data graphs in BIM so that building designers can inspect the results directly in BIM [34].

A major advantage of our approach is that Modelica is supported by a growing community of researchers who are developing various physics-based modules for simulation. Our approach is generalizable to integration of BIM to other physics-based simulations. Currently, our* BIM2BEM* approach is focused on thermal simulation. In future work, we will expand* BIM2BEM* to cover more simulation domains including daylight and photovoltaic. Moreover, we will apply our prototype to test more building types including complex buildings to enhance the* BIM2BEM* translation method and collect measured data from real-world project to validate the* BIM2BEM* approach. We will also examine more general boundary condition generating methods, for example, [6], and apply them into the system interface of* BIM2BEM*.

## Figures and Tables

**Algorithm 1:**
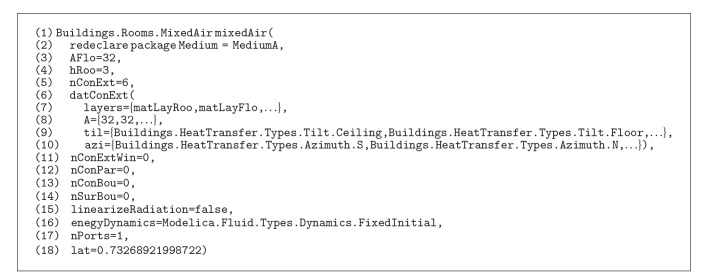
A code block for thermal zone modeling in LBNL Modelica Buildings library.

**Algorithm 2:**
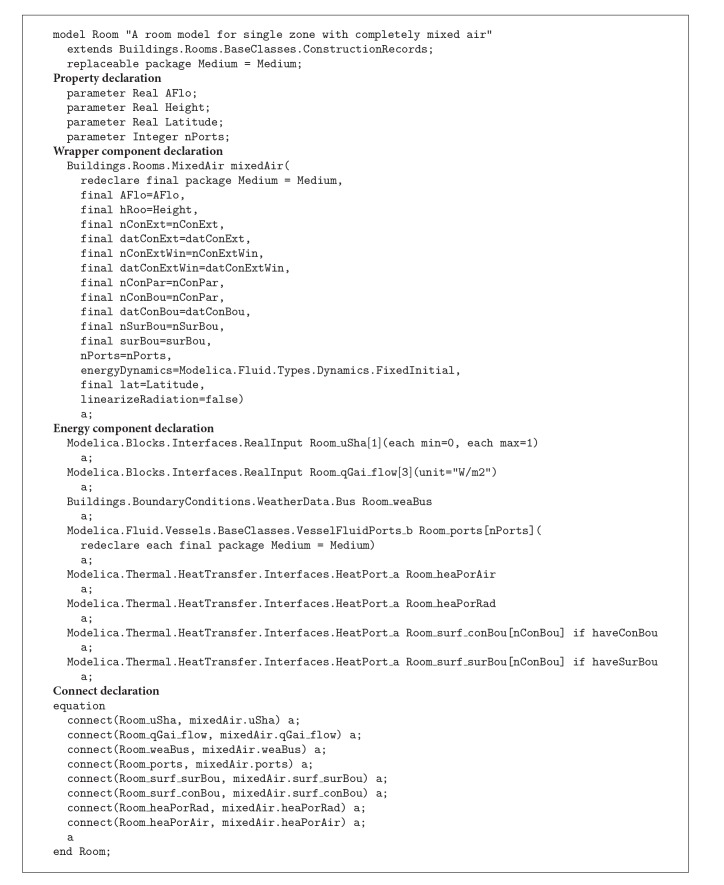
Implementation of the room class as a wrapper class based on Modelica language specification.

**Algorithm 3:**
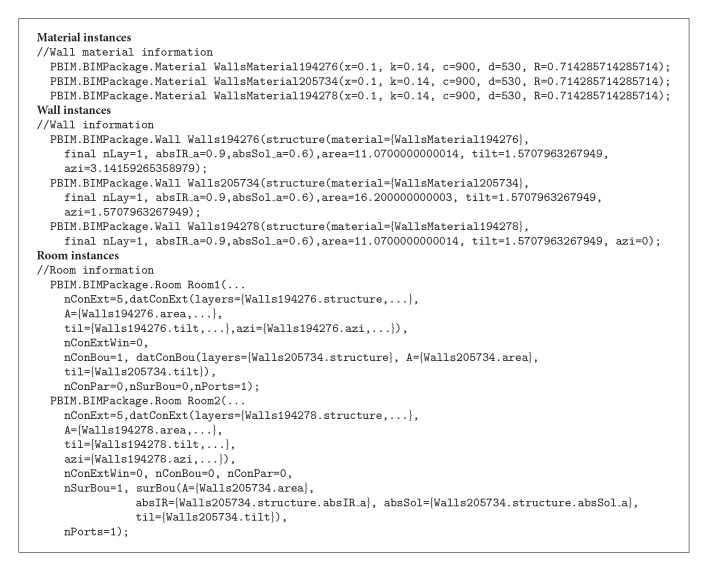
A code block of the generated *ModelicaBEM* presenting material objects for wall objects, the wall objects, and room objects using the *Revit2Modelica* prototype.

**Algorithm 4:**
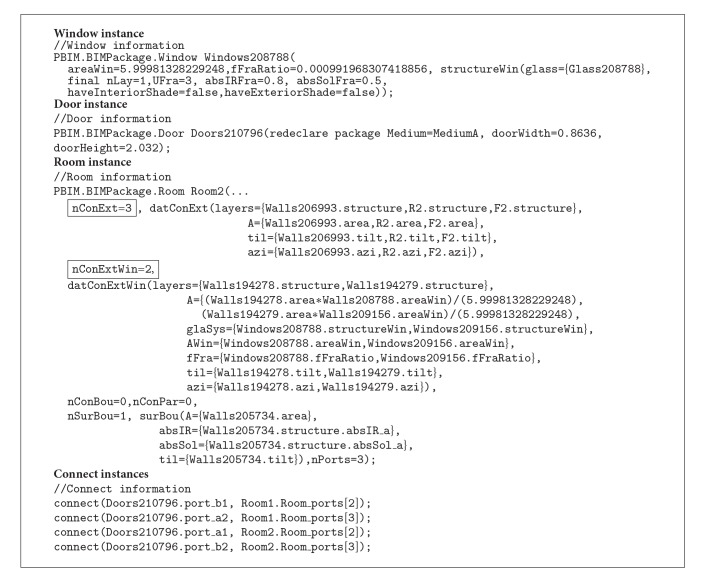
A code block of the *ModelicaBEM* presenting a window object, a door object, and connect objects of a door generated by the *Revit2Modelica* prototype.

**Algorithm 5:**
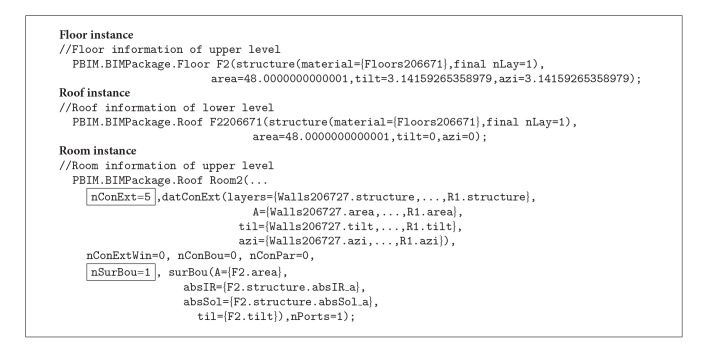
Generated *ModelicaBEM* code block of the two-story building model presenting the floor instance, the roof instance, and the room instance at the upper level.

**Table 1:** Classes and parameters adapted from LBNL Modelica Buildings library [9].

Classes	Object properties	
	Name	Description
MixedAir models a room with completely mixed air for heat transfer through a building envelop. The room consists of any number of construction types and surfaces for heat exchange through convection, conduction, and infrared radiation and solar radiation.	Medium	The medium information of a room air such as gas, most air, and dry air.
	aFlo	The floor area attached to a room.
	hRoo	The roof area attached to a room.
	datConExt	Opaque surfaces.
	nConExt	Number of datConExt.
	datConExtWin	Opaque surfaces with windows.
	nConExtWin	Number of datConExtWin.
	datConPar	Interior partitions in a thermal zone.
	nConPar	Number of datConPar.
	datConBou	Opaque surfaces on interior walls between thermal zones.
	nConBou	Number of datConBou.
	surBou	Opaque surfaces on the same interior walls between thermal zones.
	nSurBou	Number of SurBou.
	nPorts	Number of ports that constructs equations to simulate physical processes.
	Latitude	Latitude information of a room.
	energyDynamics	The information of fluid types in networks of vessels, pipes, fluid machines, vales, and fittings.
	linearizeRadiation	A setting value whether to linearize emissive power or not.

Opaque constructions describe material definitions for constructions with one or more layers of material.	matLayExt	Construction material for exterior walls.

GlazingSystem describes thermal properties for glazing systems.	nLay	Number of glass layers.
	haveExteriorShade	A setting value whether a window has an exterior shade or not.
	haveInteriorShade	A setting value whether a window has an interior shade or not.
	Glass	Thermophysical properties for window glass.
	Gas	Thermophysical properties for window gas fills.
	uFra	*U*-value of frame.
	absIRFra	Infrared absorptivity of window frame.
	absSolFra	Solar absorptivity of window frame.

DoorDiscretizedOpen describes the bidirectional airflow through an open door.	Medium	The medium information of the room airflow through an open door.
	Width	Width of opening
	Height	Height of opening

**Table 2:** Defined steps and required data.

Steps	Description	Data requirements
Create/edit BIM	Architects can create building components such as walls, floors, roofs, doors, and windows to represent their design intents and specifications in Revit.	(i) Geometry information: area, tilt, azimuth, height, and width. (ii) Material information: thickness (iii) Supplementary information: project location, identification numbers for each building components.

Define physical parameters and extra parameters	In order to map missed physical properties in Revit into LBNL Modelica Buildings library, we defined the step of adding the physical parameters, e.g., solar and infrared absorptivities, in existing material properties in Revit. Those physical parameters are added through Revit API. Additional information regarding the glazing system for thermal modeling needs to be prepared in Revit. The properties for glass thickness and ratio of window frame can be added via updating the window family, and parameters for material properties, e.g., solar transmittance, can be added by using Revit API.	(i) Additional material information: thermal conductivity, specific heat capacity, mass density, and solar and infrared absorptivities (ii) Additional thermal information for glazing system: glass thickness, the ratio of window frame (iii) Additional material information for glass: solar transmittance, infrared transmissivity of glass, solar reflectance of surface, *U*-value of frame, infrared and solar absorptivity of window frames

Assign zones	To conduct room-to-thermal zone translation, zoning information is required in Revit. We defined thermal zones by using room components in Revit. The room components basically contain the information of height and the area attached to floors. The latitude information for the room can be retrieved from a Revit function.	(i) Room information: height, area of floors, and latitude (ii) Zoning information: latitude

Create topology	The thermal information for heat transfer of the building envelope can be prepared in Revit: the information of how the building envelope is constructed, e.g., boundary condition types, and the number of ports for thermal network connections in *ModelicaBEM* can be generated based on the building topology information retrieved using Revit API. The building topology provides the information of how many and what building components are connected to a room. The information will be the values of the boundary condition variables in a MixedAir instance in *ModelicaBEM*.	(i) Thermal information: boundary condition types and the number of ports (ii) Building components information

**Table 3:** Material specification.

Building components	Thermal conductivity (W/m K)	Specific heat capacity (J/kg K)	Mass density (kg/m^3^)	Thickness (m)
Walls	0.140	900	530	0.100
Floor	0.140	1200	650	1.028
Roof	0.160	840	950	0.150

**Table 4:** Annual peak temperatures of the test cases.

Cases	Room name	Highest temperature (°C)/Date-Time	Lowest temperature (°C)/Date-Time
1	East	ModelicaBEM: 34.63°C/July 19th-6PM	ModelicaBEM: −15.479°C/February 7th-8AM
		LBNL model: 34.66°C/July 19th-6PM	LBNL model: −15.471°C/February 7th-8AM
	West	ModelicaBEM: 35.222°C/July 18th-7PM	ModelicaBEM: −15.655°C/February 7th-9AM
		LBNL model: 35.237°C/July 18th-7PM	LBNL model: −15.658°C/February 7th-9AM

2	East	ModelicaBEM: 42.827°C/August 21st-12PM	ModelicaBEM: −15.369°C/February 7th-6AM
		LBNL model: 42.968°C/August 21st-12PM	LBNL model: −15.669°C/February 7th-6AM
	West	ModelicaBEM: 35.485°C/July 18th-6PM	ModelicaBEM: −11.967°C/February 7th-8AM
		LBNL model: 35.413°C/July 18th-6PM	LBNL model: −11.955°C/February 7th-8AM

3	Upper	ModelicaBEM: 38.085°C/July 18th-6PM	ModelicaBEM: −21.289°C/January 8th-8AM
		LBNL model: 38.035°C/July 18th-6PM	LBNL model: −21.283°C/January 8th-8AM
	Lower	ModelicaBEM: 40.144°C/July 18th-2PM	ModelicaBEM: −10.348°C/February 7th-6AM
		LBNL model: 40.047°C/July 18th-2PM	LBNL model: −10.029°C/February 7th-6AM
